# Isolated absence of right pulmonary artery

**DOI:** 10.4103/0974-2069.74037

**Published:** 2010

**Authors:** Anil Kumar Singhi, Edwin Francis, Raman Krishna Kumar

**Affiliations:** Department of Cardiology, The Children Hospital at Westmead, Sydney, Australia; 1 Department of Pediatric Cardiology, Amrita Institute of Medical Sciences, Kerala, India

**Keywords:** Absent right pulmonary artery, chest X-ray, pulmonary hypertension

## Abstract

**Background:**

Absence of right pulmonary artery (RPA) is a rare congenital abnormality with variable presentation. The diagnosis is often missed in infants

**Objective:**

The aim of this study was to describe infantile presentation of isolated absence of RPA along with a brief review of the literature.

**Methods:**

The details of five patients diagnosed with isolated absence of RPA from April 2007 to October 2009 were reviewed retrospectively

**Results:**

Five patients were identified with this anomaly. The median age of presentation was 86 days (range, 40–120 days) and the median weight was 3.65 kg (range, 3.1–5.5 kg). All patients presented with breathing difficulty and had severe pulmonary hypertension (PHT) along with absent RPA on echocardiography. A multidetector computed tomographic scan was performed to confirm the diagnosis in four cases. Three patients had major aortopulmonary collateral and the hilar RPA was not well developed in all. A correct diagnosis was made before referral in one patient only. Differential vascularity in chest X-ray, a useful clue, was seen in four of five cases. Surgical correction was not considered in view of the small hilar pulmonary artery. The patients were all managed medically with diuretics and Sildenafil.

**Conclusion:**

Isolated absence of RPA is a rare congenital abnormality with varied presentation. Infantile presentation is marked with congestive cardiac failure and PHT. Specific diagnostic clue includes differential vascularity on the chest X-ray.

## INTRODUCTION

Isolated absence of RPA is a rare congenital lesion with a diverse clinical presentation. This defect was first reported by Fraentzel in 1868.[1] Pool *et al*. studied 78 cases with absence of either of the branch pulmonary arteries and found 32 cases (~40%) without any additional cardiac lesion.[2] Fifty-six patients in their series had additional structural cardiac problems such as tetralogy of Fallot (21%), patent ductus arteriosus (PDA; 14%) and septal defects (9%). Isolated absence of RPA was associated with PHT in 19-44% of the patients in different case series.[2–4]

Isolated absence of RPA frequently presents in infancy with PHT and congestive cardiac failure.[4,5] A high index of suspicion is required to diagnose this condition in infancy.[6,7] We present a series of five infants along with a brief review of the literature.

## METHODS

We retrospectively reviewed the medical records of children who presented with severe PHT from April 2007 to October 2009. Patients with isolated absence of RPA were identified and reviewed in detail. Patients associated with other congenital heart defects were excluded.

## RESULTS

**Figure 1:**
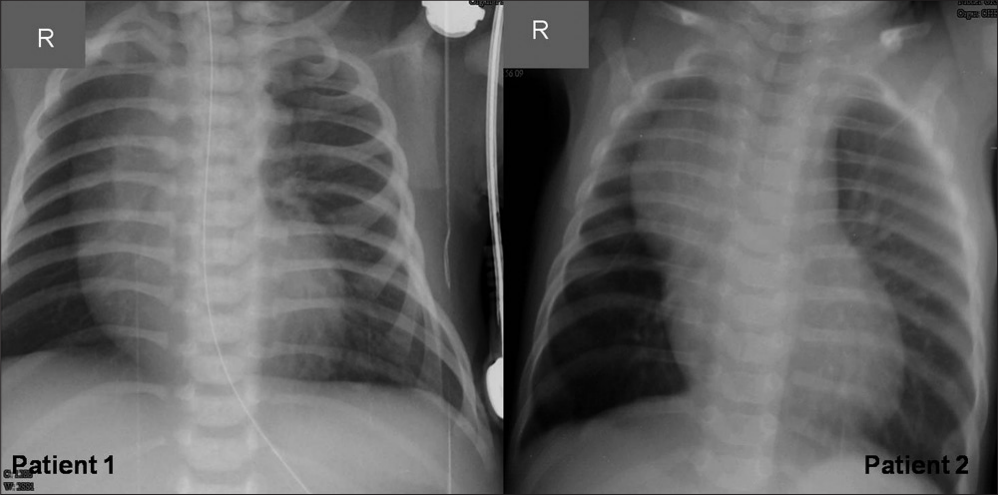
Chest X-ray (posteroanterior view) of patient# 1 and patient# 2 showing diminished hilar pulmonary artery shadow, a shrunken right lung and a shift of the mediastinal structures to the right side. Right-sided pulmonary vascular markings are diminished

In a period of two and half years, we had five patients diagnosed as isolated absence of RPA. All patients were infants between 40 and 120 days old (mean age, 85 days) at presentation, weighing 3.1-5.5 kg (mean weight, 3.99 kg). Four out of five of the patients were referred as unexplained cases of severe PHT. All infants had echocardiogram performed in the referring hospital. Only one of the patients was referred with diagnosis of absent RPA and severe PHT. The clinical presentation of the patients was similar [Table 1]. All had tachypnea at the time of presentation and had clinical features of PHT. Electrocardiogram showed sinus tachycardia, rightward QRS axis and right ventricular dominance. X-ray of the chest [Figure 1] in the posterior-anterior view showed differential vascularity in four out of the five babies, which was an important clue. Initial echocardiographic evaluation showed severe PHT and the RPA was found to be absent in all of them Table 1.

**Table 1:** Characteristics of the five patients with unilateral absence of right pulmonary artery

Patient no. #	Age in days	Weight in kg	Sex	Complete diagnosis
1	40	3.1	Male	Absent RPA, severe PAH. RVSP 70 + RAP in mmHg by TR jet. Right lung supplied by aortopulmonary collateral (only by echocardiography)
2	80	4.1	Female	Absent RPA, severe PAH. RVSP 64 + RAP in mmHg by TR jet. MAPCA from right proximal brachiocephalic trunk and descending aorta
3	120	3.6	Female	Absent RPA, severe PAH (by septal position). No evidence of MAPCAs
4	103	5.5	Male	Absent RPA, severe PAH. RVSP 60 + RAP in mmHg by TR jet. MAPCA from right subclavian and descending thoracic aorta supplying the right lung
5	86	3.65	Female	Absent RPA, severe PAH. RVSP 64 + RAP in mmHg by TR jet. MAPCA noted arising from the right subclavian artery

**Figure 2:**
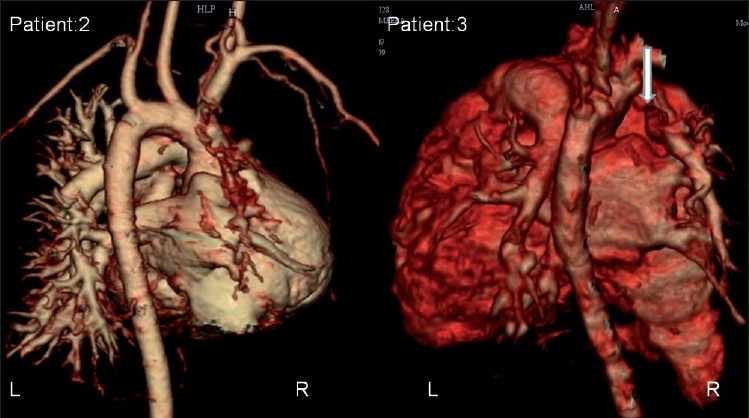
Multidetector computed tomography images of patient# 2 and patient# 3. The heart is seen from its posterior aspect. The right pulmonary artery is absent and the hilar pulmonary artery is diminutive (arrow). R, right side; L, left side

A cardiac multidetector computed tomographic (MDCT) scan was carried out in four patients to confirm the diagnosis and to define the pulmonary artery anatomy. The hilar pulmonary artery was not well developed [Figure 2]. Three patients (Patient# 2, 4 and 5) had major aortopulmonary collateral artery (MAPCA) detected by the MDCT scan. Patient# 1 had suspicion of collateral on echocardiography, but an MDCT scan was not performed. Patient# 3 had no significant collateral on the MDCT scan [Table 1]. The hilar pulmonary artery was very small in the babies and thus surgical correction was not considered. The babies were treated symptomatically with frusemide (1 mg/kg twice daily), spironolactone (2 mg/kg/day) and Sildenafil (1 mg/kg/day). On follow-up (mean 12 months), three of the four babies were symptomatically stable with decongestive medication and Sildenafil. All had persistent PHT. One patient was lost to follow-up.

## DISCUSSION

Isolated absence of RPA is a rare disease. Bouros *et al*. described the prevalence by analyzing the chest radiograph of 600,000 army recruits and found a prevalence of 1 in 200,000.[6] The study was carried out in the healthy, adult population. The true prevalence of the disease in children is not known.

The entity of unilateral absence of pulmonary artery was reviewed in three publications covering different time era in the last ~60 years. Pool *et al*.[2] reviewed 32 cases before 1962 whereas Shakibi *et al*.[4] studied 47 cases from 1962 to 1976. Hankel *et al*.[3] added a review of 107 cases from 1978 to 2000. RPA was absent in 56–63% of the cases in these three case reviews. Cucci *et al*. opined that absence of RPA was more frequently seen in an otherwise normal heart and absence of left pulmonary artery was accompanied by additional cardiac lesion.[8] This fact was not supported in the analysis of 186 cases in three series.[2–4] All the five infants in our series had absent RPA, which could be a simple coincidence.

Two types of presentations are described. The first presentation is the one seen in infants, where they usually present with congestive cardiac failure and PHT.[4,5] The other presentation is in older patients, who usually do not develop pulmonary hypertension and do not have manifest heart failure. They present with exercise intolerance (18–40%), hemoptysis (20%) or are incidentally detected during chest radiography.[4,5] Our patients fall in the first category, i.e. infants with congestive cardiac failure and PHT. The incidence of PHT among the patients with unilateral absence of pulmonary artery is reported to be between 19 and 44% in different case series.[2–4]

The diagnosis of isolated absence of RPA is based on history, clinical evaluation and imaging. A high index of suspicion is needed to make the diagnosis. In infancy, the signs can be subtle and can be easily missed.[5–7] Four of the five cases in our series were diagnosed as a case of unexplained severe PHT after echocardiography in the referring institution.

The electrocardiogram is usually normal in patients with uncomplicated isolated absence of pulmonary artery (without PHT), whereas it shows right ventricular dominance in cases associated with PHT, as seen in all the infants in our series.[3]

Chest X-ray may show an absent hilar shadow, absence of the left or RPA, reduction of pulmonary vascular markings on the right side (~60% cases), a small hemithorax and intercostal bone space, shrunken affected lung and a shift of the mediastinal structures to the affected side with contralateral lung hyperinflation.[3–5] A cardiac MDCT scan [Figure 2] and magnetic resonance imaging (MRI) can confirm the diagnosis and delineate the pulmonary artery anatomy along with delineating MAPCA if present.

Any infant with unexplained PHT should be thoroughly evaluated for the possibility of isolated unilateral absence of pulmonary artery (UAPA). Differential vascularity on the chest X-ray may often be a clue for the diagnosis. Four of the five patients in our series had diminished vascular marking on the affected side [Figure 1].

### PHT and isolated absence of the RPA

Infants with isolated absence of the one of the pulmonary arteries frequently present with PHT.[4,5] Pool *et al*. documented medial hypertrophy of the pulmonary artery of the normal lung (opposite to the affected side) in 53% of the 17 patients.[2] The affected lung (lacking a pulmonary artery) was free of these changes in 83% of the cases.[2] Haworth *et al*. described the changes in the right and left pulmonary artery after ligation of the left pulmonary artery and ductus arteriosus in the animal model (14 newborn pigs).[9,10] The muscularity of the RPA (<75 micron diameter) remained high. The left pulmonary artery and branches were small, with a disorganized elastin wall structure, although the muscularity of the arteries in the left side was reduced. The mean pulmonary artery pressure was elevated (20–35 mmHg) along with features of hypertrophied right ventricle. The persistence of a fetal structure of the contralateral pulmonary artery (opposite to the affected side) was the predominant reason cited for PHT.[9,11] Other reasons postulated for PHT were insufficient elasticity of the pulmonary vascular bed of the normal side receiving full cardiac output and abnormal response to vasoconstrictor.[11,12] Pool *et al*. opined that those patients with isolated unilateral absence of pulmonary artery who develop PHT generally do so at an early age and die from right heart failure. However, if PHT did not develop at an early age, it is unlikely that it will develop later.[2]

### Therapeutic approach

The therapeutic approach for isolated absence of right pulmonary artery should be based on symptoms of the patient, pulmonary artery anatomy and associated aortopulmonary collateral. No treatment is required in the patients without any evidence of cardiopulmonary dysfunction (as seen in adults with incidental detection). They should be followed-up on a regular basis.[4]

In patients with signs of severe congestive cardiac failure, symptomatic treatment should be offered early, such as surgical intervention that may potentially preserve the affected lung vasculature and prevent morbidity and mortality. The intrapulmonary branches of the pulmonary artery are usually present and hence surgical anastomosis is possible.[3,4,13]

If the intrapulmonary arteries are well developed, a primary anastomosis with the central pulmonary artery can be made. If the intrapulmonary branches are small, a modified Blalock-Taussig shunt can be used. This allows a better growth of the affected intrapulmonary arteries before the segments are connected with the main pulmonary artery during subsequent surgery.[7,13] Other surgical options are an interposition tube graft with autologous pericardium or prosthetic material and mobilization with end-to-side anastomosis of the affected artery to the main pulmonary artery.[14] The hilar pulmonary artery in the infants of our series (four patients) was very small and hence surgical correction was not considered.

Krammoh *et al*. described their two-stage therapeutic approach in three patients. The first stage was PDA stenting, followed by surgical anastomosis in the second stage. The patients required anticoagulation therapy after the ductal stenting. They reported good outcome in three cases during midterm follow-up.[15] Coil occlusion of MAPCA is also reported in cases with hemoptysis.[3] Patients who have delayed diagnosis and delayed surgical interventions may develop irreversible hypoplasia and regression of the affected pulmonary artery and have a less-favorable outcome.[16,17]

For patients who are not considered suitable for revascularization or when PHT does not improve, therapeutic measures for PHT may be helpful. Long-term vasodilator therapy may improve survival. Sildenafil, calcium channel blockers and continuous intravenous infusion of prostacyclin have been tried with variable response.[3] Infants who present with severe PHT, as seen in our series, represent the most difficult subset to treat. PHT in this group is not known to regress spontaneously.[5] The overall mortality across all the age groups is reported to be 7%, and infants with severe PHT have poor outcome.[3]

## CONCLUSION

Isolated absence of RPA is a rare entity. In infancy, it presents with respiratory distress and severe PHT. A high index of suspicion is needed to diagnose the entity. Differential vascularity in the chest X-ray can give a clue. MDCT scan and MRI can confirm the echocardiographic diagnosis and delineate the pulmonary artery anatomy. The surgical treatment plan depends on the presence of a good-sized hilar pulmonary artery and the presence of MAPCA. Early surgical or hybrid intervention may improve survival. Medical management includes symptomatic treatment for congestive cardiac failure and long-term pulmonary vasodilator for PHT. Infants with severe PHT are difficult to treat and have an unfavorable outcome.
